# Microeukaryotes Associated with Freshwater Mussels in Rivers of the Southeastern United States

**DOI:** 10.3390/microorganisms12091835

**Published:** 2024-09-05

**Authors:** Akacia K. Halliday-Isaac, Colin R. Jackson

**Affiliations:** Department of Biology, University of Mississippi, University, Oxford, MS 38677, USA; akhallid@go.olemiss.edu

**Keywords:** Apicomplexa, apicomplexans, freshwater mussels, microbial diversity, microeukaryotes, protists

## Abstract

Microeukaryotes are a diverse and often overlooked group of microbes that are important in food webs and other ecological linkages. Little is known about microeukaryotes associated with aquatic invertebrates, although filter feeders such as mussels are likely to take in and potentially retain microeukaryotes in their gut while feeding. Microeukaryotes such as apicomplexans have been reported in marine mussel species, but no studies have examined the presence of these microorganisms in freshwater mussels or how they relate to mussel host species or environmental conditions. In this study, microbial community DNA was extracted from the gut tissue of over 300 freshwater mussels, representing 22 species collected from rivers in the southeastern USA. Microeukaryote DNA was detected using PCR amplification, followed by the sequencing of positive amplicons. Microeukaryotes were found in 167 individual mussels (53%) of those tested. Amplicons included dinoflagellates/algae that differed between mussel species and are likely food sources that were distinct from those found in water and sediment samples analyzed concurrently. A total of 5% of the positive amplicons were non-photosynthetic alveolates that could represent parasitic microeukaryotes. Understanding the distribution of microeukaryotes in the freshwater mussel gut microbiome could further our understanding of the ongoing decline of mussel populations.

## 1. Introduction

Freshwater mussels are benthic macroinvertebrates that feed on particulate organic matter, such as bacteria, phytoplankton, and algae filtered from the water column and through interstitial sediment. These macroinvertebrates perform important ecosystem services, such as maintaining water quality, stimulating benthic-pelagic nutrient cycling, providing structural habitat, and modifying aquatic food webs [1]. Typically classified as filter feeders, filtration rates of freshwater mussels vary with species, size, and environmental conditions, and pedal feeding, where freshwater mussels use cilia on their foot to collect benthic organic matter, can also be an important source of food [2,3,4]. When coexisting as multispecies aggregates, freshwater mussels likely use resource partitioning and niche diversification to reduce interspecies competition [5,6,7], and mussels can selectively feed on specific algal taxa, with those preferences differing between mussel species [8].

North America houses a large portion of the global freshwater mussel biodiversity, primarily unionid mussels, although 70% of North American mussel species are listed as endangered, threatened, or of special concern. These mussel populations are declining because of increased pollution and changes in hydrology and climate [9]. Over the last few years, there has been increased interest in investigating the gut microbiome of North American unionid mussels [10,11,12,13] and its importance to host health and function. While such studies have shown that the gut microbiome varies spatially and temporally, as well as with mussel species, these studies have focused almost entirely on the bacterial community within the gut, even though freshwater mussels almost certainly interact with other components of the microbial community. Given that protists such as dinoflagellates are common in freshwater systems of North America [14], it seems likely that the unionid mussel gut microbiome would include eukaryotic microorganisms, as well as bacteria, and mussel feeding preferences show the consumption of eukaryotic microorganisms such as Stramenopiles, Chlorophytes, and fungi by unionid mussels [15]. Unionid mussels can also harbor eukaryotes, such as mites, trematodes, and leeches, as well as ciliates, such as Tetrahymena [16,17,18]. Parasitic taxa, including the ciliates *Conchophthirus* sp. [19] and *Trichodina* sp. [20], the trematodes *Rhipidocotyle campanula* and *Phyllodistomum* sp. [19,20,21], and mites of the *Unionicola* genus [22,23] have been documented in freshwater mussels, primarily in Europe. Other infectious agents described in freshwater mussels include neoplasms, viruses, bacteria, fungi, fungal-like agents, Aspidogastrea, Digenea, and Acari [24,25]. However, compared to European freshwater bivalves, the presence of endosymbionts in North American freshwater mussels is understudied [26].

This difference is particularly apparent among members of the ubiquitous protistan group, Apicomplexa. While freshwater unionid mussels have no documented species-specific apicomplexans, bent mussels (*Ischadium recurvum*) and zebra mussels (*Dreissena polymorpha*), both of which are associated with low salinity or freshwater environments, have been shown to take up *Cryptosporidium* from terrestrial runoff and retain them in their gut tissues [27,28]. In contrast to freshwater mussels, a number of studies have reported that marine bivalves can host microeukaryotes, particularly members of the ubiquitous protistan group, Apicomplexa. Apicomplexans are obligate intracellular protozoans found in vertebrate and invertebrate hosts in terrestrial and aquatic habitats, and the group includes the causative agents of important infectious diseases, such as malaria, cryptosporidiosis, and toxoplasmosis [29]. Marine bivalves, such as the eastern oyster (*Crassostrea virginica*), great scallop (*Pectin maximus*), and the Atlantic Sea scallop (*Plecopecten magellanicus*), have been found to contain a variety of apicomplexans from the genera *Aggregata*, *Merocystis*, *Margolisiella,* and *Nematopsis* [30,31,32], as well as an infectious sister taxon of the *Perkinsus* genus that causes mass mortality [33,34,35]. Marine bivalves can also acquire terrestrial apicomplexans, with reports of the black mussel (*Mytilus galloprovincialis*) containing oocysts of the terrestrial apicomplexan *Cryptosporidium* [36]. 

The presence of apicomplexans in a variety of other bivalves suggests the hypothesis that unionid mussels could also contain apicomplexans or other microeukaryotes. Furthermore, the complex lifestyle of freshwater unionid mussels involves their larvae encapsulating themselves on the gills or fins of freshwater fish. Such ecological interactions with fish hosts have shaped the evolutionary history of freshwater mussels [37], and spending a portion of their life on a fish host could expose larval mussels to microeukaryotes, as fish often have apicomplexans that spend a portion of their life cycle in gill tissue [38,39,40]. 

The goal of this study was to determine what microeukaryotes, if any, are associated with freshwater mussels in the southeastern United States, a global hotspot for unionid mussel biodiversity. As part of ongoing surveys looking at the freshwater mussel gut microbiome, more than 20 species of mussels were collected from rivers in the Mobile and Tennessee River basins, and we tested the gut tissue of these mussels for the presence of microeukaryotes. More specifically, this study expands on the current knowledge of apicomplexan infections in bivalves by determining (1) if apicomplexans are present in these freshwater mussels and (2) if the presence of apicomplexans correlates with specific mussel species or locations.

## 2. Materials and Methods

As part of ongoing studies on the bacterial microbiome of freshwater mussels in the southeastern United States, mussels were surveyed and collected from sites on Bear Creek, Bogue Chitto Creek, the Buttahatchee and Sipsey rivers of the Mobile River Basin, and the Duck and Paint Rock rivers of the Tennessee River Basin (see Hopper et al. [41] for more site information). Mussels were collected from July–September 2019 and processed as described by McCauley et al. [12] and Chiarello et al. [42]. Briefly, samples were flash-frozen and stored at −80 °C until the gastrointestinal tract was excised and ground; the microbial DNA was extracted using a PowerSoil Pro kit (Qiagen, Germantown, MD, USA) and stored frozen until use in this study [12,42]. At the time of collection, samples of water and sediment were also taken from each site. Water was filtered, and DNA was extracted from filters and collected sediment following the same procedures as ground mussel gut tissue. Environmental physicochemical data (temperature, pH, conductivity, ammonia, nitrates, nitrites, and dissolved oxygen) were recorded over the sampling period.

This study used gut community DNA extracted from 22 mussel species (311 mussel gut DNA samples total) collected in the previous studies: *Amblema plicata*, *Cyclonaias tuberculata*, *Elliptio arca*, *Elliptio crassidens*, *Fusconaia cerina*, *Hamiota perovalis*, *Lampsilis ornata*, *Lampsilis ovata*, *Lampsilis teres*, *Lasmigona alabamensis*, *Lasmigona costata*, *Megalonaias nervosa*, *Obliquaria reflexa*, *Obovaria unicolor*, *Pleurobema oviforme*, *Potamilus purpuratus*, *Ptychobranchus fasciolaris*, *Pustulosa kieneriana* (formerly *Cyclonaias asperata* [37]), *Pustulosa pustulosa* (formerly *Cyclonaias pustulosa* [37]), *Quadrula quadrula* [formerly *Quadrula apiculata* and *Quadrula rumphiana* [43]), *Quadrula verrucosa* (formerly *Tritogonia verrucosa* [37]), and *Toxolasma lividum*. DNA was also used from the environmental samples (sediment and water) collected from the same sample sites.

To screen for microeukaryotes, a region of the 18S small subunit ribosomal DNA (SSU rDNA) was amplified using a primer pair designed for apicomplexans SFC-340f (5′-AGTTTCTGACCTATCAGC-3′) and SFC-1260r (5′-TCAGCCTTGCGACCATACTC-3′) [44]. PCR was performed in 20 µL reactions using OneTaq 2X Master Mix (New England Biolabs, Ipswich, MA, USA), 0.5 µM forward and reverse primer concentrations, and approximately 20 ng/µL of template DNA. Amplification involved an initial denaturing step of 5 min at 95 °C, followed by 35 cycles of 94 °C for 30 s, 55 °C for 45 s, and 72 °C for 1 min. The procedure ended with a terminal extension of 72 °C for 7 min. 

The presence/absence of amplification products was checked with agarose gel electrophoresis. Positive amplicons were sequenced at Functional Biosciences, Inc. (Madison, WI, USA), and trace files were edited using MEGA11 [45]. Sequences were identified to the family and genus levels using the Silva database https://www.arb-silva.de/aligner/ (accessed on 12 February 2024) and NCBI Blast in December 2023. Positive sequencing results were separated into sequences that were classified as dinoflagellates/algae or non-photosynthetic alveolates (potentially Apicomplexa). In addition to the sequences generated in this study (sequence accession numbers PP964320-PP964486), representative Apicomplexa and dinoflagellate sequences from GenBank were included as references (Supplemental Material, Table S1). 

An SSU rDNA phylogenetic tree was generated analyzing the non-photosynthetic alveolates generated in this study, along with 14 representative Apicomplexa, *Perkinsus marinus* (AF324218), and *Chromera velia* (DQ174731), as an outgroup. These sequences were from GenBank (Supplementary Material, Table S2). Sequences were aligned in MEGA11 [45] using MUSCLE [46] with default parameters and alignments, followed by manual checks. The best model was selected by ModelFinder and implemented in IQ-Tree version 1.6.12. The maximum likelihood tree was generated using nonparametric bootstrapping and 1000 ultrafast bootstrap iterations in IQ-Tree. The resulting tree used an HKY+F+R3 model. The annotation of the ML tree was performed using Interactive Tree of Life [47].

A binomial logistic regression was used to investigate the relationship between the presence of non-photosynthetic alveolates and mussel phylogeny (tribe and subfamily), the number of reproductive hosts (as derived from [48]), and environmental physicochemical variables (temperature, pH, conductivity, ammonia, nitrates, nitrites, and dissolved oxygen). A multinominal logistic regression was used to investigate the relationship between the presence of dinoflagellate/algal amplicons, mussel species, mussel life history traits, and collection site. The multinomial logistic regression was performed using the “nnet” package version 7.3-19 [49] in R version 4.2.2 [50]. To confirm patterns, a generalized linear model was performed, with the proportion of amplicon presences for individuals of a mussel species at a site as the response and mussel species, mussel life history traits, environmental physicochemical variables (temperature, pH, conductivity, ammonia, nitrates, nitrites, and dissolved oxygen), and collection site as predictor variables. 

## 3. Results

Of the 311 samples of DNA from the gut tissue of freshwater mussels tested for amplification with the microeukaryote primer set, 167 (53.7%) showed positive amplifications that could be identified following sequencing. The positive amplifications came from DNA extracted from 20 of the 22 species of freshwater mussels examined, with only gut DNA from *Lasmigona costata* and *Obovaria unicolor* yielding no amplification products. Sequencing the 167 amplicons yielded sequences that averaged 840 bases long and then were trimmed to 700 bases after initial sequence processing and editing. The majority (158, or 94.6%, of the total) of these sequences were identified as dinoflagellates or algae, and nine (5.4%) were identified as non-photosynthetic alveolates. Five sequences were unclassifiable due to low match similarity. 

The most frequently detected dinoflagellate/algal sequences were identified as being from the genera *Monodus*, *Trachydiscus*, and *Unruhdinium*. Mussel species varied in the dinoflagellate or algal sequences amplified from their gut DNA, and species that were sampled from multiple rivers yielded different amplicons from different rivers (Table 1). 

**Figure 1:**
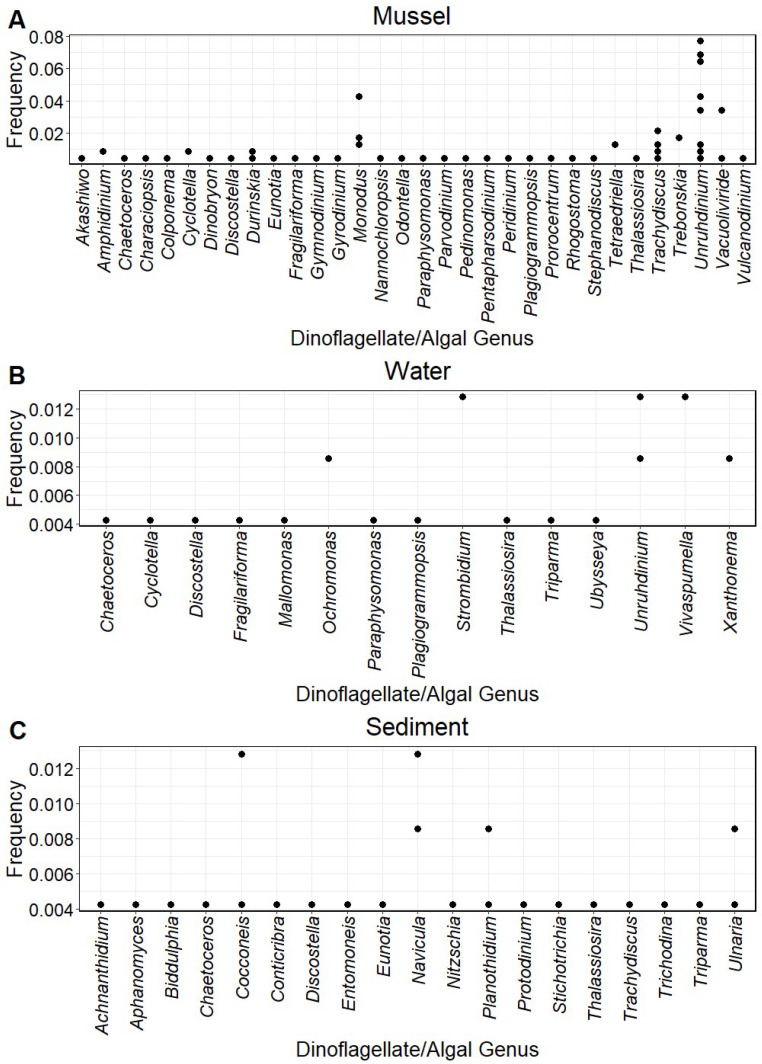
Frequency of 18S rDNA dinoflagellate and algal amplicons obtained from the guts of freshwater mussels ((**A**); n = 158), filtered water ((**B**); n = 35), and sediment ((**C**); n = 34) collected from rivers in the Mobile and Tennessee River basins. Frequency indicates how frequently the amplicon occurred in samples analyzed, and each point represents the frequency of an amplicon at a sample site or within a specific mussel species sampled from a site.

*Unruhdinium* were the most frequently identified microeukaryotic taxa, with 78 amplicons. Bogue Chitto Creek and the Paint Rock River had the greatest variety of amplicons. The amplicons present and their frequency differed among the species *Elliptio arca* (*p* = 0.000519), *Hamiota perovalis* (*p* = 0.001651), *Lampsilis ornata* (*p* = 0.000519), *Lampsilis teres* (*p* = 0.011303), *Lasmigona alabamensis* (*p* = 0.011303), *Megalonaias nervosa* (*p* = 0.000520), *Obliquaria reflexa* (*p* = 0.001651), *Potamilus purpuratus* (*p* = 0.000520), *Pustulosa kieneriana* (*p* = 0.040035), *Quadrula quadrula* (*p* = 0.011303), and *Quadrula verrucosa* (*p* = 0.011038). However, amplicon presence did not correlate with any host tribe, subfamily, and the number of reproductive hosts (*p* > 0.05 for all). Amplicons sequenced from mussel gut tissue included more genera than those from the water and sediment collected from the same sites. The presence of amplicons in mussel samples was more similar to that in water samples than sediment (Figure 1). 

Nine sequences were identified as non-photosynthetic alveolates (Table 2). Of these, four came from mussels collected from the Paint Rock River, three came from the Sipsey River, and one each came from Bogue Chitto Creek and the Buttahatchee River. Mussel species yielding these non-photosynthetic alveolate amplicons were *Pustulosa kieneriana* (three specimens), *Pleurobema oviforme* (two specimens), *Quadrula verrucosa* (two), and one sample each from *Lampsilis ovata* and *Ptychobranchus fasciolaris*. The DNA from non-photosynthetic taxa amplified were identified as apicomplexans from the genera *Ascogregarina*, *Cryptosporidium*, *Goussia*, *Gregarina* (all class Conoidasida), and *Babesia* (class Aconoidasida), as well as other protists of the genera *Rhogostoma* (class Thecofilosea) and *Blastocystis* (class Blastocystea) (Table 2). No non-photosynthetic alveolate DNA was amplified from water or sediment samples. The presence of non-photosynthetic taxa in mussel gut DNA positively correlated with mussel species, temperature, conductivity, dissolved oxygen, nitrite, and orthophosphate. Presence was negatively correlated with ammonia, nitrate, and pH (*p* < 0.001 for all; χ² (47) = −298.30, Pseudo-R² (Cragg-Uhler) = −19.17, Pseudo-R² (McFadden) = −4.80, AIC = 456.44, BIC = 599.98).

**Figure 2:**
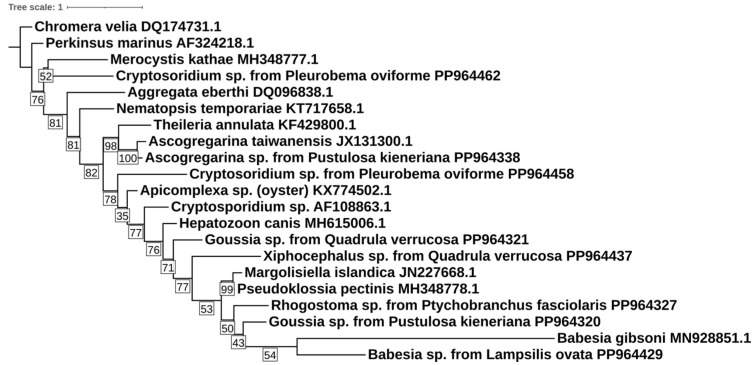
Phylogenetic analysis of 18S rDNA non-photosynthetic alveolate amplicons obtained from the gut tissue of freshwater mussels collected from rivers in the Mobile and Tennessee River basins. The phylogenetic tree was generated using an HKY+F+R3 in IQ-TREE. Bootstrap values are displayed in the translucent white box on the branches.

A further phylogenetic analysis of the alveolate sequences showed low congruence between the identified amplicons and their closest expected relatives on the tree (Figure 2). For example, you would expect the Cryptosporidium amplicons to be close together, as well as groups like Theleria and Babesia.

## 4. Discussion

In addition to a diverse community of gut bacteria [10,11,12,13], freshwater unionid mussels have been shown to harbor microscopic eukaryotes, including nematodes, trematodes, mites, fungi, and ciliated protists [16,24,25]. The role of these microorganisms in disease is unclear, and their presence may be transitory, as some opportunistic consumption of fungi by mussels may occur [15]. Unlike their marine counterparts, freshwater mussels have not been examined for the presence of protists within Phylum Apicomplexa. This study used molecular approaches (amplification with specific 18S small ribosomal subunit primers, followed by amplicon sequencing) to characterize apicomplexan and other microeukaryotes in gut DNA recovered from 22 species of unionid mussels collected from six rivers in the southeastern United States.

Sequences identified as dinoflagellate or algal taxa (i.e., photosynthetic microeukaryotes) were the most common amplicons detected in DNA from the gut tissue of freshwater mussels, being found in just over half of the >300 samples examined. Photosynthetic microeukaryotes are unlikely to be resident members of the gut microbial community, so these taxa potentially represent at least part of the mussel’s food source. The amplicons detected differed by mussel species, even between species collected from the same site or river. While some of those differences may be due to chance (only one amplicon was sequenced per mussel sample), the detection of different dinoflagellate/algal DNA in the guts of different species of freshwater mussels aligns with the idea that freshwater mussels show research partitioning and species-specific food source preferences [7,8], especially in mixed-species assemblages, such as the communities from which these mussels were sampled [42]. 

The most frequently detected sequences in the amplicons from mussel gut tissue were identified as belonging to the genera *Monodus*, *Trachydiscus*, *Unruhdinium*, and *Vacuoliviride*. *Unruhdinium* is a genus of freshwater dinoflagellates found in lakes and rivers that can form high biomass blooms [51], while *Monodus*, *Trachydiscus*, and *Vacuoliviride* are all the genera of the Eustigmatophyceae lineage of algae that live primarily in freshwater [52]. Of these four most commonly detected photosynthetic microeukaryotes, only *Unruhdinium* was identified in amplicons sequenced from samples of water or sediment, suggesting that microalgae in the guts of freshwater mussels do not just reflect those of the most common taxa in the surrounding environment, something that has also been shown for their gut bacteria [10]. Detected algae varied with mussel species but was not correlated to any life history traits, including host size range. This finding of algae preference varying solely by species is similar to what was seen in previous studies of resource partitioning [8]. Previous works evaluating food selection and resource partitioning in freshwater mussels were performed via particle size [53,54]. Sequencing of DNA extracted from gut contents or tissue was used to assess dietary preferences of freshwater fish [55], as well as marine invertebrates, such as lobster [56] and bivalves [57]. Our results support the idea that that the same approach could be used to more thoroughly examine the feeding preferences of freshwater mussel species. 

Nine of the amplicons were identified as non-photosynthetic alveolates. These sequences were all identified as representatives of parasitic phyla, mostly from the phylum Apicomplexa. These included two sequences (from DNA extracted from a *Pustulosa kieneriana* and a *Quadrula verrucosa* obtained from the Sipsey River) identified as members of the genus *Goussia*, which parasitize freshwater and marine fish [58,59,60], a sequence of the genus *Babesia*, and two sequences (both in DNA extracted from specimens of *Pleurobema oviforme* collected from the Paint Rock River) identified as *Cryptosporidium*, a waterborne parasite of livestock and humans that can also infect other wildlife [61,62]. *Cryptosporidium* showed low prevalence in a recent survey of freshwater fauna, being found in only 2/74 samples of freshwater fish and none in almost 300 aquatic insect larvae tested [63]. The high-volume filter-feeding behavior of bivalves potentially makes them more likely to take up apicomplexan oocysts than other macroinvertebrates, and *Cryptosporidium* has been shown to be taken up from terrestrial runoff by both bent mussels and zebra mussels [27,28].

The *Goussia* sequence amplified from a sample of *Quadrula verrucosa* (PP964321) was 98.45% similar to an isolate from *Goussia pannonica*, a parasite of the white bream (*Blicca bjoerkna*) [63], and the *Goussia* sequence amplicon from *Pustulosa kieneriana* (PP964320) was 92.03% identical to an isolate of *Goussia bayae*, a parasite of the white perch (*Morone americana*) [64]. The *Babesia* amplicon obtained from a *Lampsilis ovata* (PP964429) was 97.17% identical to *Babesia gibsoni*, a tick-borne parasite that infects terrestrial mammals. This sequence is, however, closer to the *Psuedoklossia* and *Margosiella* sequences in the phylogeny, suggesting that this sequence could be unique. A likely route of entrance into freshwater systems could be runoff, similar to what has been seen with the genus *Cryptosporidium.* While having no cystic life stages, a related terrestrial apicomplexan that infects livestock (*Theileria* spp.) has been seen to infect electric eels in Brazil [64]. It is possible that unionid mussels could be exposed to parasites such as *Goussia* and *Babesia,* as their larvae feed on the blood of fish while attaching to their gills. This warrants further exploration. 

Of the nine samples that tested positive for apicomplexans, four were from DNA extracted from the guts of mussels collected from the Paint Rock River, and three were from DNA from mussels from the Sipsey River. Studies have shown that factors such as water temperature, ammonia, and total nitrogen induce stress and correlate with reduced density in mussel populations [65,66]. In this study, incidence positively correlated with temperature, conductivity, dissolved oxygen, nitrite, and orthophosphate, while it negatively correlated with ammonia, nitrate, and pH. However, while water chemistry and physiology correlated with alveolate incidence, the low frequency of alveolates makes these relationships unclear. Protist infections in other marine bivalves are thought to be related to stress caused by environmental factors [67], so this is worth further exploration. 

Only one amplicon per mussel sample was sequenced during this study, limiting our ability to identify less common microeukaryotes. The majority of sequences obtained were photosynthetic microeukaryotes (dinoflagellates, algae) that, as potential food sources, are likely to be more prevalent in the guts of unionid mussels than apicomplexans or other non-food taxa. It is quite possible that more of the samples we examined contained apicomplexans, but these were not detected with our approach, which was designed more as a broad survey of multiple mussel species rather than focusing on identifying specific potential parasites. However, even with our limited approach, almost 3% (9/311) of the samples that were tested yielded positive amplicons that were identified as apicomplexan DNA, similar to the numbers reported for the presence of *Cryptosporidium* in a survey of freshwater fish [63]. Further work should use more in-depth sequencing of individual mussel hosts, potentially using high-throughput sequencing, as has been used on the bacterial gut microbiome. The low bootstrap values and variable placement of the non-photosynthetic alveolate amplicons within the phylogenetic analysis indicate that the tree is fluid, reflecting our still-emerging knowledge on the diversity of apicomplexans. The presence of potentially unique Apicomplexa, such as the Babesia amplicon, signals the need to more specifically explore potential host-parasite interactions between apicomplexans and unionid mussels, and the development and application of a more specific apicomplexan primer set designed for that purpose would help offer a better understanding of their diversity. 

While molecular studies on the gut bacterial community associated with freshwater mussels are increasing [10,12,42], fewer have considered the microeukaryotes that may also be associated with freshwater mussels. Most of the eukaryotic sequences we detected were identified as algae or dinoflagellates and potentially represent the food source of those mussels. However, we also detected eukaryotic sequences that were likely from parasitic taxa. While some uptake of apicomplexans in poor water conditions were previously shown in some freshwater bivalves [27,28], the presence of apicomplexans in freshwater unionid mussels has not been previously examined. Unionid mussels are declining in North America [68,69,70], and host-microeukaryote interactions could be exacerbating the current stressors to the population. The declines being seen can be exacerbated by the presence of harbored microscopic eukaryotes. While little research has been performed to support the idea, the decline of North American mussels has been suggested as being at least partly due to an unknown pathogen [71], so the presence of parasitic apicomplexans in some of the mussel samples we examined merits further investigation.

## Figures and Tables

**Table 1:** Dinoflagellate and algae amplicons from freshwater mussel gut tissue. There were 20 species of mussels with identified amplicons. River indicates sampling location: Bear Creek (Bear), Bogue Chitto Creek (Bogue), the Buttahatchee (Butta) and Sipsey rivers of the Mobile River Basin, and the Duck and Paint Rock (Paint) rivers of the Tennessee River Basin. “No.” shows the number of amplicons identified as the indicated genera from the mussel species and river. Identity is the % identity match to NCBI database sequences.

Mussel Species	River	Microeukaryote Amplicon Phlyum:Family:Genus	No.	Identity
*Amblema* *plicata*	Bear	Ochrophyta: Paraphysomonadaceae: *Paraphysomonas*	1	89.8
	Bogue	Dinophyceae: Kryptoperidiniaceae: *Unruhdinium*	4	89.0–99.1
		Ochrophyta: Eunotiaceae: *Eunotia*	1	83.6
		Ochrophyta: Fragilariaceae: *Fragilariforma*	1	94.6
		Ochrophyta: Stephanodiscaceae: *Stephanodiscus*	1	80.2
		Ochrophyta: Pleurochloridaceae: *Monodus*	2	91.6–95.0
	Duck	Dinophyceae: Kryptoperidiniaceae: *Unruhdinium*	1	99.6
		Heterokontophyta:Cymatosiraceae: *Plagiogrammopsis*	1	99.6
	Paint	Dinophyceae:Kryptoperidiniaceae: *Unruhdinium*	5	81.3–95.5
	Sipsey	Dinophyceae: Kryptoperidiniaceae: *Unruhdinium*	1	99.7
*Cyclonaias* *tuberculata*	Duck	Heterokontophyta: Stephanodiscaceae: *Cyclotella*	1	93.4
	Paint	Chlorophyta: Pedinomonadaceae: *Pedinomonas*	1	96.5
		Dinophyceae: Amphidiniaceae *Amphidinium*	1	81.6
		Dinophyceae: Kryptoperidiniaceae: *Unruhdinium*	9	97.2–99.8
		Dinophyceae: Peridiniopsidaceae: *Parvodinium*	1	81.4
*Elliptio* *arca*	Butta	Heterokontophyta: Goniochloridaceae: *Tetraedriella*	2	84.2–94.7
		Heterokontophyta: Goniochloridaceae: *Trachydiscus*	1	92.5
	Sipsey	Dinophyceae: Gymnodiniaceae: *Akashiwo*	1	73.3
*Elliptio* *crassidens*	Bear	Ochrophyta: Goniochloridaceae: *Trachydiscus*	1	88.72
		Dinophyceae: Kryptoperidiniaceae: *Unruhdinium*	2	88.1–96.3
*Fusconaia* *cerina*	Butta	Ochrophyta: Pleurochloridaceae: *Monodus*	1	95.5
		Ochrophyta: Monodopsidaceae: *Nannochloropsis*	1	87
		Heterokontophyta: Goniochloridaceae: *Trachydiscus*	2	82.7–92.5
*Hamiota* *perovalis*	Sipsey	Dinophyceae: Kryptoperidiniaceae: *Unruhdinium*	1	83.6
*Lampsilis* *ornata*	Bogue	Heterokontophyta: Pleurochloridaceae: *Monodus*	2	92.6–93.3
		Ochrophyta: Cymatosiraceae: *Plagiogrammopsis*	1	85.12
		Ochrophyta: Goniochloridaceae: *Trachydiscus*	1	86.31
	Butta	Heterokontophyta: Pleurochloridaceae: *Monodus*	2	90.9–94.9
	Sipsey	Dinophyceae: Kryptoperidiniaceae: *Unruhdinium*	1	93.93
*Lampsilis* *ovata*	Bear	Dinophyceae: Kryptoperidiniaceae: *Unruhdinium*	4	85.9–99.4
		Ochrophyta: Goniochloridaceae: *Trachydiscus*	1	91.6
	Paint	Colponemida: Colponemidia: *Colponema*	1	96.6
		Dinophyceae: Kryptoperidiniaceae: *Unruhdinium*	9	82.8–99.6
		Dinophyceae: Kryptoperidiniaceae: *Durinskia*	2	83.3–88.0
		Dinophyceae: Prorocentraceae: *Prorocentrum*	1	90.3
*Lampsilis* *teres*	Bogue	Ochrophyta: Goniochloridaceae: *Trachydiscus*	1	87.2
		Ochrophyta: Pleurochloridaceae: *Monodus*	1	92.5
*Lasmigona* *alabamensis*	Bogue	Dinophyceae: Kryptoperidiniaceae: *Unruhdinium*	1	99.5
		Ochrophyta: Thalassiosiraceae: *Thalassiosira*	1	86.3
*Megalonaias* *nervosa*	Bogue	Dinophyceae: Kryptoperidiniaceae: *Unruhdinium*	1	87.8
*Obliquaria* *reflexa*	Bogue	Dinophyceae: Kryptoperidiniaceae: *Unruhdinium*	1	86.0
	Duck	Dinophyceae: Kryptoperidiniaceae: *Unruhdinium*	1	98.9
		Heterokontophyta: Stephanodiscaceae: *Cyclotella*	1	99.8
	Sipsey	Heterokontophyta: Goniochloridaceae: *Vacuoliviride*	1	98.8
*Pleurobema* *oviforme*	Paint	Dinophyceae: Kryptoperidiniaceae: *Durinskia*	1	99.2
		Dinophyceae: Kryptoperidiniaceae: *Unruhdinium*	4	98.8–99.1
*Potamilus* *purpuratus*	Bogue	Ochrophyta: Pleurochloridaceae: *Monodus*	1	91.5
*Ptychobranchus* *fasciolaris*	Paint	Dinophyceae: Kryptoperidiniaceae: *Unruhdinium*	2	97.9–99.2
		Ochrophyta: Stephanodiscaceae: *Discostella*	1	87.5
		Ochrophyta: Thalassiosiraceae: *Thalassiosira*	1	88.9
*Pustulosa* *kieneriana*	Bogue	Dinophyceae: Kryptoperidiniaceae: *Unruhdinium*	6	84.2–97.7
		Ochrophyta: Chlorobotryaceae: *Characiopsis*	1	84.9
		Ochrophyta: Cymatosiraceae: *Plagiogrammopsis*	1	88.9
		Ochrophyta: Dinobryaceae: *Dinobryon*	1	96.3
		Ochrophyta: Goniochloridaceae: *Vacuoliviride*	1	93
		Ochrophyta: Monodopsidaceae: *Nannochloropsis*	1	90.4
		Ochrophyta: Pleurochloridaceae: *Monodus*	3	84.1–87.1
	Butta	Heterokontophyta: Goniochloridaceae: *Tetraedriella*	1	92.7
		Heterokontophyta: Goniochloridaceae: *Trachydiscus*	1	94.7
		Ochrophyta: Pleurochloridaceae: *Monodus*	1	93.5
	Sipsey	Heterokontophyta: Goniochloridaceae: *Trachydiscus*	1	97.9
		Ochrophyta: Goniochloridaceae: *Vacuoliviride*	1	98.7
*Pustulosa* *pustulosa*	Bear	Dinophyceae: Kryptoperidiniaceae: *Unruhdinium*	15	81.3–98.9
		Heterokontophyta: Goniochloridaceae: *Trachydiscus*	1	98.5
		Ochrophyta: Stephanodiscaceae: *Discostella*	1	98.5
	Duck	Heterokontophyta: Chaetocerotaceae: *Chaetoceros*	1	96.6
		Dinophyceae: Kryptoperidiniaceae: *Unruhdinium*	1	98.8
	Paint	Dinophyceae: Amphidiniaceae: *Amphidinium*	1	84.8
		Dinophyceae: Kryptoperidiniaceae: *Unruhdinium*	2	92.9–97.3
		Dinophyceae: Peridiniales incertae sedis: *Vulcanodinium*	1	85.1
*Quadrula* *quadrula*	Bogue	Ochrophyta: Pleurochloridaceae: *Monodus*	4	89.9–96.3
		Dinophyceae: Kryptoperidiniaceae: *Unruhdinium*	3	91.9–98.9
*Quadrula* *verrucosa*	Butta	Heterokontophyta: Goniochloridaceae: *Trachydiscus*	3	87.2–96.46
	Paint	Dinophyceae: Kryptoperidiniaceae: *Unruhdinium*	3	98.7–99.5
	Sipsey	Heterokontophyta: Goniochloridaceae: *Trachydiscus*	4	85.8–92.9
		Heterokontophyta: Goniochloridaceae: *Trebonskia*	2	89.4–95.4
		Dinophyceae: Kryptoperidiniaceae: *Unruhdinium*	2	92.3–95.5
*Toxolasma* *lividum*	Paint	Dinophyceae: Ensiculiferaceae: *Pentapharsodinium*	1	88.1
		Dinophyceae: Kryptoperidiniaceae: *Unruhdinium*	3	87.5–98.7
		Heterokontophyta: Chaetocerotaceae: *Chaetoceros*	1	84.2

**Table 2:** Alveolate amplicons from freshwater mussel gut tissues. Mussels were collected from Bogue Chitto Creek (Bogue), the Buttahatchee River (Butta), the Sipsey River in the Mobile River Basin, and the Paint Rock River (Paint) in the Tennessee River Basin. A total of 22 mussel species were tested, and only those yielding positive amplicons identified as non-photosynthetic microeukaryotes are shown. No. indicates the number of amplicons identified as the indicated genera from the mussel species and river. Identity is the % identity match to NCBI database sequences.

Mussel Species	River	Amplicon Phlyum:Family:Genus	No.	Identity
*Lampsilis* *ovata*	Paint	Apicomplexa: Babesiidae: *Babesia*	1	94.6
*Pleurobema* *oviforme*	Paint	Apicomplexa: Cryptosporidiidae: *Cryptosporidium*	2	87.0–90.3
*Ptychobranchus* *fasciolaris*	Paint	Cercozoa: Rhogostomidae: *Rhogostoma*	1	91.1
*Pustulosa* *kieneriana*	Sipsey	Apicomplexa: Lecudinidae: *Ascogregarina*	1	91.3
		Bigyra: Blastocystidae: *Blastocystis*	1	80.8
	Bogue	Apicomplexa: Barrouxiidae: *Goussia*	1	91.8
*Quadrula* *verrucosa*	Butta	Apicomplexa: Gregarinidae: *Gregarina*	1	88.7
	Sipsey	Apicomplexa: Barrouxiidae: *Goussia*	1	98.4

## Data Availability

The sequences generated in this study were uploaded to Genbank under the accession numbers PP964320-PP964486.

## Supplementary Materials

The following supporting information can be downloaded at: https://www.mdpi.com/article/10.3390/microorganisms12091835/s1, Table S1: GenBank accession numbers and metadata of sequences; Table S2: The taxa and respective accession numbers for the 18S rRNA gene sequence used in this study.
