# Supplementary material for: Microeukaryotes Associated with Freshwater Mussels in Rivers of the Southeastern United States
Source: Microorganisms. 2024 Sep 5;12(9):1835. doi: 10.3390/microorganisms12091835 (PMC11434547; doi:10.3390/microorganisms12091835)
Supplement: Supplementary file 1 [file microorganisms-12-01835-s001.zip › microorganisms-3113665-supplementary.docx]

Table S1. GenBank Accession numbers and metadata of sequences. River indicates sampling location: Bear Creek (Bear), Bogue Chitto Creek(Bogue), the Buttahatchee (Butta) and Sipsey rivers of the Mobile River Basin, and the Duck and Paint Rock (Paint) rivers in the Tennessee River basin. The genus identified and the % sequence homology was determined using NCBI Blast in December 2023

| Sample ID | Basin | River | Species | % homology to NCBI sequence | Genus Identity | Genbank Accession Number |
| --- | --- | --- | --- | --- | --- | --- |
| MDB0001 | Mobile | Sipsey | *Pustulosa kieneriana* | 91.89 | Goussia | PP964320 |
| MDB0002 | Mobile | Sipsey | *Quadrula verrucosa* | 98.35 | Goussia | PP964321 |
| MDB0005 | Mobile | Bogue | *Lampsilis teres* | 87.23 | Trachydiscus | PP964322 |
| MDB0006 | Mobile | Sipsey | *Amblema plicata* | 93.55 | Unruhdinium | PP964323 |
| MDB0007 | Mobile | Bogue | *Lasmigona alabamensis* | 99.48 | Unruhdinium | PP964324 |
| MDB0008 | Mobile | Bogue | *Lasmigona alabamensis* | 86.25 | Thalassiosira | PP964325 |
| MDB0009 | Mobile | Bogue | *Megalonaias nervosa* | 87.79 | Unruhdinium | PP964326 |
| MDB0010 | Tennessee | Paint | *Ptychobranchus fasciolaris* | 90.83 | Rhogostoma | PP964327 |
| MDB0011 | Tennessee | Paint | *Ptychobranchus fasciolaris* | 97.92 | Unruhdinium | PP964328 |
| MDB0012 | Tennessee | Paint | *Ptychobranchus fasciolaris* | 88.96 | Thalassiosira | PP964329 |
| MDB0013 | Tennessee | Paint | *Ptychobranchus fasciolaris* | 99.2 | Unruhdinium | PP964330 |
| MDB0014 | Tennessee | Paint | *Ptychobranchus fasciolaris* | 87.54 | Discostella | PP964331 |
| MDB0015 | Mobile | Bogue | *Pustulosa kieneriana* | 97.75 | Unruhdinium | PP964332 |
| MDB0016 | Mobile | Bogue | *Pustulosa kieneriana* | 90.24 | Unruhdinium | PP964333 |
| MDB0019 | Mobile | Bogue | *Pustulosa kieneriana* | 84.23 | Unruhdinium | PP964334 |
| MDB0021 | Mobile | Bogue | *Pustulosa kieneriana* | 84.13 | Monodus | PP964335 |
| MDB0022 | Mobile | Bogue | *Pustulosa kieneriana* | 84.86 | Monodus | PP964336 |
| MDB0023 | Mobile | Bogue | *Pustulosa kieneriana* | 91.69 | Unruhdinium | PP964337 |
| MDB0024 | Mobile | Bogue | *Pustulosa kieneriana* | 91.34 | Ascogregarina | PP964338 |
| MDB0025 | Mobile | Bogue | *Pustulosa kieneriana* | 97.71 | Unruhdinium | PP964339 |
| MDB0026 | Mobile | Bogue | *Quadrula quadrula* | 89.93 | Monodus | PP964340 |
| MDB0027 | Mobile | Bogue | *Quadrula quadrula* | 98.25 | Unruhdinium | PP964341 |
| MDB0028 | Mobile | Bogue | *Quadrula quadrula* | 95.9 | Monodus | PP964342 |
| MDB0030 | Mobile | Bogue | *Quadrula quadrula* | 91.97 | Unruhdinium | PP964343 |
| MDB0033 | Mobile | Bogue | *Quadrula quadrula* | 96.26 | Monodus | PP964344 |
| MDB0034 | Mobile | Bogue | *Quadrula quadrula* | 94.32 | Monodus | PP964345 |
| MDB0035 | Mobile | Bogue | *Quadrula quadrula* | 98.98 | Unruhdinium | PP964346 |
| MDB0036 | Mobile | Bogue | *Amblema plicata* | 88.96 | Unruhdinium | PP964347 |
| MDB0037 | Mobile | Bogue | *Amblema plicata* | 99.06 | Unruhdinium | PP964348 |
| MDB0038 | Mobile | Bogue | *Amblema plicata* | 91.58 | Monodus | PP964349 |
| MDB0039 | Mobile | Bogue | *Amblema plicata* | 94.64 | Fragilariforma | PP964350 |
| MDB0040 | Mobile | Bogue | *Amblema plicata* | 94.97 | Monodus | PP964351 |
| MDB0041 | Mobile | Bogue | *Amblema plicata* | 83.52 | Unruhdinium | PP964352 |
| MDB0042 | Mobile | Bogue | *Amblema plicata* | 83.57 | Eunotia | PP964353 |
| MDB0043 | Mobile | Bogue | *Amblema plicata* | 79.19 | Odontella | PP964354 |
| MDB0044 | Mobile | Bogue | *Amblema plicata* | 93.86 | Unruhdinium | PP964355 |
| MDB0046 | Mobile | Bogue | *Potamilus purpuratus* | 91.54 | Monodus | PP964356 |
| MDB0047 | Mobile | Bogue | *Obliquaria reflexa* | 86.01 | Unruhdinium | PP964357 |
| MDB0048 | Mobile | Bogue | *Lampsilis ornata* | 85.12 | Plagiogrammopsis | PP964358 |
| MDB0049 | Mobile | Bogue | *Lampsilis teres* | 92.5 | Monodus | PP964359 |
| MDB0050 | Mobile | Bogue | *Pustulosa kieneriana* | 96.32 | Dinobryon | PP964360 |
| MDB0054 | Mobile | Bogue | *Pustulosa kieneriana* | 88.92 | Plagiogrammopsis | PP964361 |
| MDB0055 | Mobile | Bogue | *Pustulosa kieneriana* | 87.13 | Monodus | PP964362 |
| MDB0056 | Mobile | Bogue | *Pustulosa kieneriana* | 83.74 | Unruhdinium | PP964363 |
| MDB0057 | Mobile | Bogue | *Pustulosa kieneriana* | 84.99 | Characiopsis | PP964364 |
| MDB0059 | Mobile | Bogue | *Pustulosa kieneriana* | 93.04 | Vacuoliviride | PP964365 |
| MDB0060 | Mobile | Bogue | *Pustulosa kieneriana* | 90.37 | Nannochloropsis | PP964366 |
| MDB0061 | Mobile | Bogue | *Lampsilis ornata* | 86.31 | Trachydiscus | PP964367 |
| MDB0063 | Mobile | Bogue | *Lampsilis ornata* | 92.61 | Monodus | PP964368 |
| MDB0064 | Mobile | Bogue | *Lampsilis ornata* | 93.31 | Monodus | PP964369 |
| MDB0065 | Mobile | Bear | *Pustulosa pustulosa* | 99.36 | Unruhdinium | PP964370 |
| MDB0066 | Mobile | Bear | *Pustulosa pustulosa* | 99.12 | Unruhdinium | PP964371 |
| MDB0067 | Mobile | Bear | *Pustulosa pustulosa* | 99.62 | Unruhdinium | PP964372 |
| MDB0068 | Mobile | Bear | *Pustulosa pustulosa* | 98.86 | Unruhdinium | PP964373 |
| MDB0071 | Mobile | Bear | *Pustulosa pustulosa* | 99.74 | Unruhdinium | PP964374 |
| MDB0072 | Mobile | Bear | *Pustulosa pustulosa* | 99.1 | Unruhdinium | PP964375 |
| MDB0073 | Mobile | Bear | *Pustulosa pustulosa* | 96.32 | Unruhdinium | PP964376 |
| MDB0074 | Mobile | Bear | *Pustulosa pustulosa* | 99.24 | Unruhdinium | PP964377 |
| MDB0075 | Mobile | Bear | *Pustulosa pustulosa* | 87.91 | Unruhdinium | PP964378 |
| MDB0076 | Mobile | Bear | *Pustulosa pustulosa* | 81.33 | Unruhdinium | PP964379 |
| MDB0077 | Mobile | Bear | *Pustulosa pustulosa* | 98.58 | Trachydiscus | PP964380 |
| MDB0078 | Mobile | Bear | *Pustulosa pustulosa* | 80 | Stephanodiscus | PP964381 |
| MDB0079 | Mobile | Bear | *Pustulosa pustulosa* | 82.77 | Plagiogrammopsis | PP964382 |
| MDB0080 | Mobile | Bear | *Amblema plicata* | 86.55 | Paraphysomonas | PP964383 |
| MDB0083 | Mobile | Bear | *Lampsilis ovata* | 91.55 | Trachydiscus | PP964384 |
| MDB0084 | Mobile | Bear | *Lampsilis ovata* | 89.59 | Unruhdinium | PP964385 |
| MDB0085 | Mobile | Bear | *Lampsilis ovata* | 85.9 | Unruhdinium | PP964386 |
| MDB0086 | Mobile | Bear | *Lampsilis ovata* | 99.46 | Unruhdinium | PP964387 |
| MDB0088 | Mobile | Bear | *Lampsilis ovata* | 87.97 | Unruhdinium | PP964388 |
| MDB0090 | Mobile | Bear | *Elliptio crassidens* | 88.72 | Trachydiscus | PP964389 |
| MDB0092 | Mobile | Bear | *Elliptio crassidens* | 96.34 | Unruhdinium | PP964390 |
| MDB0094 | Mobile | Bear | *Elliptio crassidens* | 88.11 | Unruhdinium | PP964391 |
| MDB0095 | Mobile | Bear | *Pustulosa pustulosa* | 90.41 | Unruhdinium | PP964392 |
| MDB0097 | Mobile | Bear | *Pustulosa pustulosa* | 90.34 | Discostella | PP964393 |
| MDB0102 | Mobile | Bear | *Pustulosa pustulosa* | 87.41 | Unruhdinium | PP964394 |
| MDB0103 | Mobile | Bear | *Pustulosa pustulosa* | 80 | Prorocentrum | PP964395 |
| MDB0135 | Mobile | Sipsey | *Hamiota perovalis* | 83.73 | Akashiwo | PP964396 |
| MDB0136 | Tennessee | Paint | *Cyclonaias tuberculata* | 98.81 | Unruhdinium | PP964397 |
| MDB0138 | Tennessee | Paint | *Cyclonaias tuberculata* | 99.36 | Unruhdinium | PP964398 |
| MDB0139 | Tennessee | Paint | *Cyclonaias tuberculata* | 99.87 | Unruhdinium | PP964399 |
| MDB0141 | Tennessee | Paint | *Cyclonaias tuberculata* | 99.61 | Unruhdinium | PP964400 |
| MDB0142 | Tennessee | Paint | *Cyclonaias tuberculata* | 96.73 | Unruhdinium | PP964401 |
| MDB0143 | Tennessee | Paint | *Cyclonaias tuberculata* | 99.1 | Unruhdinium | PP964402 |
| MDB0144 | Tennessee | Paint | *Cyclonaias tuberculata* | 99.75 | Unruhdinium | PP964403 |
| MDB0145 | Tennessee | Paint | *Cyclonaias tuberculata* | 99.62 | Unruhdinium | PP964404 |
| MDB0146 | Tennessee | Paint | *Amblema plicata* | 99.35 | Unruhdinium | PP964405 |
| MDB0151 | Tennessee | Paint | *Amblema plicata* | 99.74 | Unruhdinium | PP964406 |
| MDB0152 | Tennessee | Paint | *Amblema plicata* | 97.85 | Unruhdinium | PP964407 |
| MDB0153 | Tennessee | Paint | *Amblema plicata* | 98.37 | Unruhdinium | PP964408 |
| MDB0156 | Tennessee | Paint | *Lampsilis ovata* | 82.86 | Unruhdinium | PP964409 |
| MDB0157 | Tennessee | Paint | *Lampsilis ovata* | 96.15 | Unruhdinium | PP964410 |
| MDB0158 | Tennessee | Paint | *Lampsilis ovata* | 97.59 | Unruhdinium | PP964411 |
| MDB0159 | Tennessee | Paint | *Lampsilis ovata* | 99.61 | Unruhdinium | PP964412 |
| MDB0160 | Tennessee | Paint | *Lampsilis ovata* | 99.22 | Unruhdinium | PP964413 |
| MDB0161 | Tennessee | Paint | *Pustulosa pustulosa* | 97.26 | Unruhdinium | PP964414 |
| MDB0162 | Tennessee | Paint | *Pustulosa pustulosa* | 85.07 | Vulcanodinium | PP964415 |
| MDB0163 | Tennessee | Paint | *Pustulosa pustulosa* | 84.81 | Amphidinium | PP964416 |
| MDB0164 | Tennessee | Paint | *Pustulosa pustulosa* | 92.91 | Unruhdinium | PP964417 |
| MDB0167 | Tennessee | Paint | *Toxolasma lividum* | 88.13 | Pentapharsodinium | PP964418 |
| MDB0170 | Tennessee | Paint | *Toxolasma lividum* | 87.48 | Unruhdinium | PP964419 |
| MDB0171 | Tennessee | Paint | *Toxolasma lividum* | 84.15 | Chaetoceros | PP964420 |
| MDB0173 | Tennessee | Paint | *Toxolasma lividum* | 98.72 | Unruhdinium | PP964421 |
| MDB0175 | Tennessee | Paint | *Toxolasma lividum* | 90.29 | Unruhdinium | PP964422 |
| MDB0176 | Tennessee | Paint | *Cyclonaias tuberculata* | 97.22 | Unruhdinium | PP964423 |
| MDB0177 | Tennessee | Paint | *Cyclonaias tuberculata* | 81.57 | Amphidinium | PP964424 |
| MDB0178 | Tennessee | Paint | *Cyclonaias tuberculata* | 81.4 | Parvodinium | PP964425 |
| MDB0181 | Tennessee | Paint | *Cyclonaias tuberculata* | 100 | Pedinomonas | PP964426 |
| MDB0183 | Tennessee | Paint | *Lampsilis ovata* | 96.55 | Colponema | PP964427 |
| MDB0184 | Tennessee | Paint | *Lampsilis ovata* | 83.3 | Durinskia | PP964428 |
| MDB0185 | Tennessee | Paint | *Lampsilis ovata* | 94.56 | Babesia | PP964429 |
| MDB0186 | Tennessee | Paint | *Lampsilis ovata* | 98.84 | Unruhdinium | PP964430 |
| MDB0187 | Tennessee | Paint | *Lampsilis ovata* | 90.31 | Prorocentrum | PP964431 |
| MDB0188 | Tennessee | Paint | *Lampsilis ovata* | 99.1 | Unruhdinium | PP964432 |
| MDB0189 | Tennessee | Paint | *Lampsilis ovata* | 88.01 | Durinskia | PP964433 |
| MDB0192 | Mobile | Butta | *Fusconaia cerina* | 92.54 | Trachydiscus | PP964434 |
| MDB0194 | Mobile | Butta | *Fusconaia cerina* | 87.01 | Nannochloropsis | PP964435 |
| MDB0217 | Mobile | Butta | *Quadrula verrucosa* | 94.74 | Trachydiscus | PP964436 |
| MDB0218 | Mobile | Butta | *Quadrula verrucosa* | 88.47 | Xiphocephalus | PP964437 |
| MDB0231 | Mobile | Butta | *Pustulosa kieneriana* | 94.72 | Trachydiscus | PP964438 |
| MDB0232 | Mobile | Butta | *Pustulosa kieneriana* | 93.55 | Monodus | PP964439 |
| MDB0239 | Mobile | Butta | *Pustulosa kieneriana* | 92.71 | Tetraedriella | PP964440 |
| MDB0243 | Mobile | Butta | *Elliptio arca* | 94.74 | Tetraedriella | PP964441 |
| MDB0247 | Mobile | Butta | *Elliptio arca* | 84.22 | Tetraedriella | PP964442 |
| MDB0248 | Mobile | Butta | *Elliptio arca* | 97.72 | Trachydiscus | PP964443 |
| MDB0251 | Mobile | Butta | *Fusconaia cerina* | 82.73 | Trachydiscus | PP964444 |
| MDB0253 | Mobile | Butta | *Fusconaia cerina* | 95.51 | Monodus | PP964445 |
| MDB0267 | Mobile | Butta | *Quadrula verrucosa* | 87.22 | Trachydiscus | PP964446 |
| MDB0268 | Mobile | Butta | *Quadrula verrucosa* | 96.46 | Trachydiscus | PP964447 |
| MDB0270 | Mobile | Butta | *Lampsilis ornata* | 90.91 | Monodus | PP964448 |
| MDB0277 | Mobile | Butta | *Lampsilis ornata* | 94.94 | Monodus | PP964449 |
| MDB0362 | Mobile | Sipsey | *Elliptio arca* | 100 | Gymnodinium | PP964450 |
| MDB0366 | Mobile | Sipsey | *Quadrula verrucosa* | 92.26 | Unruhdinium | PP964451 |
| MDB0381 | Mobile | Sipsey | *Lampsilis ornata* | 93.93 | Unruhdinium | PP964452 |
| MDB0494 | Tennessee | Paint | *Quadrula verrucosa* | 98.98 | Unruhdinium | PP964453 |
| MDB0498 | Tennessee | Paint | *Quadrula verrucosa* | 98.7 | Unruhdinium | PP964454 |
| MDB0508 | Tennessee | Paint | *Amblema plicata* | 87.44 | Unruhdinium | PP964455 |
| MDB0519 | Tennessee | Paint | *Lampsilis ovata* | 99.1 | Unruhdinium | PP964456 |
| MDB0521 | Tennessee | Paint | *Lampsilis ovata* | 99.1 | Unruhdinium | PP964457 |
| MDB0523 | Tennessee | Paint | *Pleurobema oviforme* | 90.32 | Cryptosoridium | PP964458 |
| MDB0525 | Tennessee | Paint | *Pleurobema oviforme* | 99.23 | Durinskia | PP964459 |
| MDB0526 | Tennessee | Paint | *Pleurobema oviforme* | 98.97 | Unruhdinium | PP964460 |
| MDB0526b | Tennessee | Paint | *Pleurobema oviforme* | 84.1 | Peridinium | PP964461 |
| MDB0528 | Tennessee | Paint | *Pleurobema oviforme* | 86.96 | Cryptosoridium | PP964462 |
| MDB0531 | Tennessee | Paint | *Pleurobema oviforme* | 98.85 | Unruhdinium | PP964463 |
| MDB0531b | Tennessee | Paint | *Pleurobema oviforme* | 89.63 | Fibrocapsa | PP964464 |
| MDB0536 | Tennessee | Duck | *Pustulosa pustulosa* | 98.85 | Unruhdinium | PP964465 |
| MDB0540 | Tennessee | Duck | *Pustulosa pustulosa* | 96.61 | Chaetoceros | PP964466 |
| MDB0546 | Tennessee | Duck | *Obliquaria reflexa* | 99.87 | Cyclotella | PP964467 |
| MDB0548 | Tennessee | Duck | *Obliquaria reflexa* | 98.99 | Unruhdinium | PP964468 |
| MDB0557 | Tennessee | Duck | *Cyclonaias tuberculata* | 96.47 | Cyclotella | PP964469 |
| MDB0566 | Tennessee | Duck | *Amblema plicata* | 77.96 | Plagiogrammopsis | PP964470 |
| MDB0567 | Tennessee | Duck | *Amblema plicata* | 99.61 | Unruhdinium | PP964471 |
| MDB0575 | Mobile | Sipsey | *Pustulosa kieneriana* | 97.97 | Trachydiscus | PP964472 |
| MDB0577 | Mobile | Sipsey | *Pustulosa kieneriana* | 98.7 | Vacuoliviride | PP964473 |
| MDB0580 | Mobile | Sipsey | *Obliquaria reflexa* | 98.84 | Vacuoliviride | PP964474 |
| MDB0603 | Mobile | Sipsey | *Quadrula verrucosa* | 95.51 | Unruhdinium | PP964475 |
| MDB0604 | Mobile | Sipsey | *Quadrula verrucosa* | 89.46 | Unruhdinium | PP964476 |
| MDB0605 | Mobile | Sipsey | *Quadrula verrucosa* | 85.88 | Trachydiscus | PP964477 |
| MDB0606 | Mobile | Sipsey | *Quadrula verrucosa* | 95.45 | Trebonskia | PP964478 |
| MDB0608 | Mobile | Sipsey | *Quadrula verrucosa* | 89.45 | Trebonskia | PP964479 |
| MDB0610 | Mobile | Sipsey | *Quadrula verrucosa* | 91.56 | Trachydiscus | PP964480 |
| MDB0611 | Mobile | Sipsey | *Quadrula verrucosa* | 89.12 | Trachydiscus | PP964481 |
| MDB0612 | Mobile | Sipsey | *Quadrula verrucosa* | 92.92 | Trachydiscus | PP964482 |
| MDB0623 | Mobile | Sipsey | *Lampsislis ornata* | 95.66 | Vacuoliviride | PP964483 |
| MDB0624 | Mobile | Sipsey | *Lampsislis ornata* | 96.45 | Vacuoliviride | PP964484 |
| MDB0625 | Mobile | Sipsey | *Lampsislis ornata* | 95.76 | Vacuoliviride | PP964485 |
| MDB0626 | Mobile | Sipsey | *Lampsislis ornata* | 90.08 | Vacuoliviride | PP964486 |

Table S2. The taxa and respective accession numbers for the 18S rRNA gene sequence used in this study.

| **Taxa** | **Accession Number** |
| --- | --- |
| Aggregata eberthi | DQ096838.1 |
| Alveolata sp. | HM245049.1 |
| Ascogregarina taiwanensis | JX131300.1 |
| Babesia gibsoni | MN928851 |
| Apicomplexa sp. | KX774502.1 |
| Cryptosporidium sp. | AF108863.1 |
| Goussia ameliae | KP411007.1 |
| Hepatozoon canis | MH615006.1 |
| Margolisiella islandica | JN227668.1 |
| Merocystis kathae | MH348777.1 |
| Nematopsis temporariae | KT717658.1 |
| Perkinsus marinus | AF324218.1 |
| Pseudoklossia pectinis | MH348778.1 |
| Rhogostoma cylindrica | KY905096.1 |
| Theileria annulata | KF429800.1 |
| Toxoplasma gondii | L49390.1 |
